# Impact of SGLT2-Inhibitor Therapy on Survival in Patients with Transthyretin Amyloid Cardiomyopathy: Analysis of a Prospective Registry Study

**DOI:** 10.3390/jcm13195966

**Published:** 2024-10-08

**Authors:** Nora Schwegel, Christina Toferer, David K. Zach, Viktoria Santner, Viktoria Höller, Jakob Lugitsch, Markus Wallner, Johannes Gollmer, Faisal Aziz, Dirk von Lewinski, Ewald Kolesnik, Klemens Ablasser, Andreas Zirlik, Harald Sourij, Nicolas Verheyen

**Affiliations:** 1 Division of Cardiology, University Heart Center Graz, Medical University of Graz, 8036 Graz, Austria; 2 Trials Unit for Interdisciplinary Metabolic Medicine, Division of Endocrinology and Diabetology, Department of Internal Medicine, Medical University of Graz, 8036 Graz, Austria

**Keywords:** transthyretin amyloid cardiomyopathy, Sodium–glucose co-transporter 2 inhibitors, survival, heart failure therapy

## Abstract

**Background:** Patients with transthyretin amyloid cardiomyopathy (ATTR-CM) represent a high-risk heart failure population with continued unmet therapeutic needs. Sodium–glucose co-transporter 2 inhibitors (SGLT2i) improve cardiovascular outcomes in patients with heart failure across the whole spectrum of ejection fraction, and first evidence regarding their safety and effectiveness in patients with ATTR-CM is arising. This study investigates the association between SGLT2i therapy and clinical outcomes in these patients. **Methods:** This is an analysis of a prospective registry conducted at a referral centre for hypertrophic cardiomyopathies including 116 patients with confirmed ATTR-CM. Fifty-one patients (44%) were treated with SGLT2i while 65 patients (56%) remained SGLT2i-naïve. **Results:** During a median follow-up of 2.6 (1.7–3.7) years, 38 patients (33%) died, of whom 11 patients (9%) received SGLT2i treatment and 27 patients (23%) were treatment-naïve. SGLT2i therapy was significantly associated with lower mortality (HR 0.457, 95%CI 0.227–0.922, *p* = 0.029). This association persisted after adjusting for age and sex (HR 0.479, 95%CI 0.235–0.977, *p* = 0.043) and after additional adjustment for eGFR, NT-proBNP, LVEF, and concomitant therapy with tafamidis (HR 0.328, 95%CI 0.141–0.760, *p* = 0.009). However, when potential immortal time bias was considered, this association lost statistical significance (HR 1.075, 95%CI 0.524–2.206, *p* = 0.843). No significant associations between SGLT2i therapy and worsening heart-failure hospitalization or cardiovascular mortality were observed. **Conclusions:** In crude analysis, SGLT2i therapy associates with better survival in patients with ATTR-CM. However, after adjustment for immortal time, this association becomes statistically insignificant. Hence, to draw final conclusions on the effectiveness of SGLT2i therapy in these patients, a randomized controlled trial is warranted.

## 1. Background and Aims

Transthyretin amyloid cardiomyopathy (ATTR-CM) is caused by an accumulation of amyloid fibrils in the myocardial extracellular space and leads to progressive heart failure. Once thought to be a rare disease, advances in non-invasive diagnostic approaches and rising awareness have led to an increase in diagnoses of ATTR-CM over the past decade [1]. Although several promising disease-modifying therapies are emerging [2,3,4], a lack of evidence remains on the beneficial effects of conventional heart failure therapy in this disease. Sodium–glucose co-transporter 2 inhibitors (SGLT2i) have been shown to reduce the development and progression of heart failure across the whole spectrum of ejection fraction, leading to the implementation of SGTL2i as a mainstay of heart-failure therapy [5,6,7,8,9,10]. However, these clinical trials excluded patients with ATTR-CM. Recently published studies indicate that SGLT2i therapy is also a safe and well-tolerated therapeutic option in patients with heart failure due to ATTR-CM, with beneficial effects on volume status, diuretic resistance, and arrhythmias [11,12,13,14]. First studies investigating the possible effects of SGLT2i treatment on clinical outcomes in these patients are arising, but evidence is sparse [15]. The present study aims to evaluate the effects of SGLT2 inhibition on top of established confounders regarding mortality and major heart-failure outcomes in patients with ATTR-CM.

## 2. Methods

### 2.1. Study Rationale and Study Population

**Figure 1:**
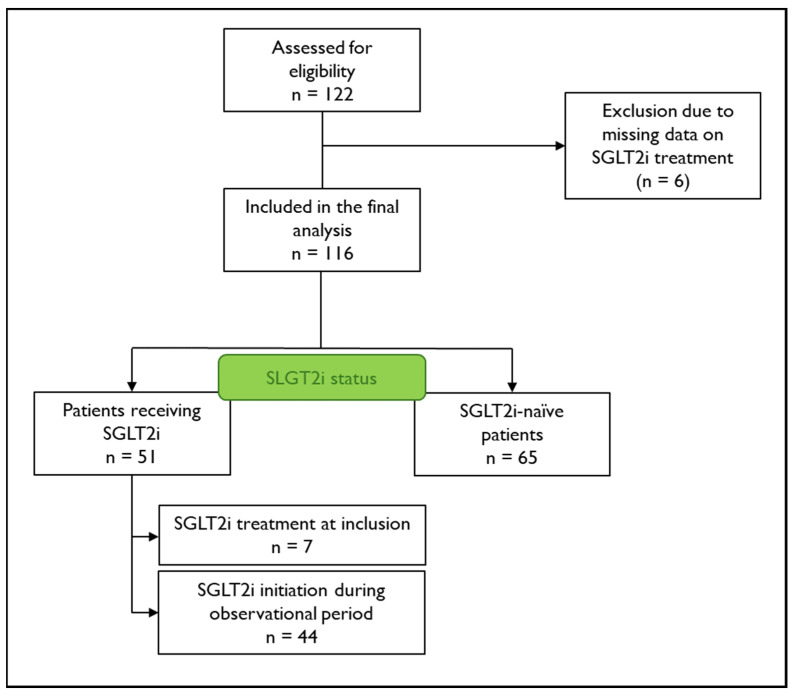
Flow chart of patient disposition. Patient disposition and Sodium–glucose co-transporter 2 inhibitor (SGLT2i) treatment status of included patients.

This is a longitudinal analysis of the prospective Graz Hypertrophic Cardiomyopathy (HCM) Registry, including all participants with a confirmed diagnosis of ATTR-CM who were enrolled between February 2019 and December 2022 (n = 122). ATTR-CM was diagnosed according to international recommendations based on confirmation by amyloid scintigraphy in combination with free light chain assessment or, in unclear cases, tissue organ biopsy [16]. Prior and concomitant SGLT2i treatment was systematically assessed according to the patient interview and medical records. Six participants (5%) were excluded from the analysis if data on SGLT2i treatment was not available, leading to a number of 116 patients eligible for analysis (Figure 1). All patients provided written informed consent. Approval was granted by the local ethics committee (EC-No. 30–286 ex 17/18), and the study was conducted in compliance with Good Clinical Practice and the Declaration of Helsinki.

### 2.2. Laboratory Parameters

Blood samples were collected at baseline in each patient. Routine laboratory parameters were immediately determined at the Clinical Institute of Medical and Chemical Laboratory Diagnostics of the Medical University of Graz, Austria.

### 2.3. Follow-Up

Patient outcomes (hospitalization due to worsening heart failure [WHF], cardiovascular death, and all-cause mortality) were retrieved from medical and health insurance records. WHF was defined as unscheduled hospitalization or unscheduled urgent outpatient clinic visit due to documented heart-failure signs and symptoms in need of initiation or significant up-titration of heart-failure treatment [17].

### 2.4. Statistical Analysis

Categorical variables were described as counts with percentages; continuous variables were expressed as a median with an interquartile range. Group comparison was performed using the Wilcoxon rank–sum for continuous variables and Chi-square or Fischer’s Exact tests for categorical variables. Associations between treatment and clinical outcomes were assessed using Cox proportional-hazard analysis with adjustment for parameters considered clinically significant confounders, namely, estimated glomerular filtration rate (eGFR), N-terminal pro-brain natriuretic peptide (NT-proBNP), left-ventricular ejection fraction (LVEF), and concomitant therapy with the selective transthyretin stabilizer tafamidis, either at baseline (SGLT2i-naïve group) or at SGLT2i initiation (SGLT2i-therapy group). All assumptions were satisfied for the reported Cox-regression models. A time-dependent Cox-regression analysis with SGLT2i treatment as time-varying exposure was performed to account for the so-called immortal time bias, which refers to the treatment-naïve time interval of patients within the treatment group (i.e., the interval between baseline examination and treatment initiation). Specifically, the follow-up time for each outcome was split before and after SGLT2i treatment initiation using the built-in function of Stata, and the Cox model was fitted on these data for each outcome, considering SGLT2i treatment as a time-varying covariate. For sensitivity analysis, landmark analysis was performed, considering year one and year two as landmark time periods to account for the immortal time bias. The steps of the analysis are provided in the supplementary material (Supplementary Figure S1). All statistical analyses were performed in Stata (Version 18.0, Stata Corp; College Station, TX, USA), considering a *p*-value < 0.05 as statistically significant.

## 3. Results

**Figure 2:**
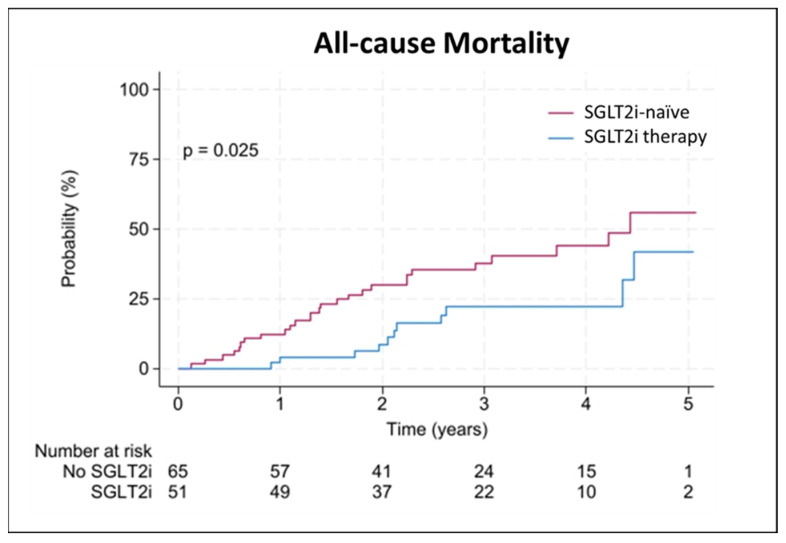
Associations between SGLT2i therapy and all-cause mortality. Kaplan Meier plot illustrating event probability for all-cause mortality with (blue) and without (red) sodium-glucose co-transporter 2 inhibitor (SGLT2i) therapy.

A total of 116 patients (15% females) were included in the final analysis, with a median (interquartile range) age of 80 (76–82) years. Hereditary ATTR-CM was present in 3% of the patients, and 15% had concomitant treatment with tafamidis at baseline. Most patients (39%) presented with New York Heart Association (NYHA) class III at baseline; angina pectoris was present in 31% of the patients. Seven patients (6%) were treated with SGLT2i at inclusion, and 44 patients (38%) were started on SGLT2i therapy during the observational period, while 65 patients (56%) remained SGLT2i-naïve. The mean (±standard deviation) treatment time with SGLT2i was 1.55 ± 0.75 years. From those receiving SGLT2i treatment, 28 patients (55%) were treated with dapagliflozin (10 mg daily), and 23 patients (45%) were treated with empagliflozin (10 mg daily). Recorded indications for SGLT2i prescription were as follows: heart failure (63%), heart failure and chronic kidney disease (18%), diabetes mellitus (8%), chronic kidney disease (6%), and heart failure and diabetes mellitus (5%). Twenty-three patients (20%) had an established diagnosis of diabetes mellitus, of whom 10 patients (9%) received SGLT2i, and 13 patients (11%) did not. LVEF at baseline was 49 (43–52)% in those who received treatment and 54 (49–59) % in the treatment-naïve group (*p* < 0.001), as shown in Table 1. At the time of treatment initiation, LVEF was 46 (39–53)%, and 30 patients (59%) were simultaneously treated with tafamidis. During the observational period, 103 patients (89%) did receive treatment with tafamidis. Of those receiving SGLT2i therapy, 48 patients (94%) were co-treated with tafamidis; of those who remained SGLT2i-naïve, 55 patients (85%) received tafamidis (*p* = 0.107, Table 2). During a median follow-up of 2.6 (1.7–3.7) years, 38 patients (33%) died, of whom 11 patients (9%) received SGLT2i treatment and 27 patients (23%) were treatment-naïve (*p* = 0.023). Fourteen deaths (12%) were classified due to a cardiovascular cause (4 patients [3%] receiving SGLT2i versus 10 [9%] SGLT2i-naïve patients; *p* = 0.216). Thirty-two patients (28%) had a WHF event, of whom 18 patients (16%) were in the treatment group, and 14 patients (22%) did not receive treatment (*p* = 0.463, see Table 3). Overall and annual incidence rates are provided in Supplementary Table S1. In univariate Cox-regression analysis, SGLT2i treatment was significantly associated with lower mortality (hazard ratio [HR] 0.457, 95% confidence interval [CI] 0.227–0.922, *p* = 0.029), as shown in Figure 2. No significant association was found with cardiovascular death (HR 1.239, 95% CI 0.654–2.346, *p* = 0.511) or WHF (HR 1.715, 95% CI 0.844–3.484, *p* = 0.136). The association between SGLT2i and lower mortality persisted after adjusting for age, sex, eGFR, NT-proBNP, LVEF, and concomitant therapy with tafamidis, either at baseline or treatment initiation time (HR 0.177, 95% CI 0.062–0.504, *p* = 0.001), as shown in Table 4. However, under consideration of a potential immortal time bias, the benefit did not remain statistically significant (HR 1.075, 95% CI 0.524–2.206, *p* = 0.843; Table 5). Detailed analyses are described in Supplementary Tables S2 and S3. No patient discontinued treatment, and no drug-related severe adverse events were reported.

## 4. Discussion

The present study shows that in patients with heart failure and a diagnosis of ATTR-CM, SGLT2i therapy is significantly associated with a lower mortality. This effect remained significant after adjustment for eGFR, NT-proBNP, LVEF, and concomitant tafamidis therapy. However, after adjustment for immortal time, the association became neutral.

In line with our results, previous reports support the notion that SGLT2i is safe and overall well-tolerated in patients with ATTR-CM [11,12,13,14]. Previous large-scale trials on the use of SGLT2i treatment in patients with heart failure also substantiate the benefit of these drugs on major heart-failure outcomes in patients with heart failure regardless of systolic function [5,6,7,8]. While ATTR-CM was an exclusion criterion in these trials, large meta-analyses indicate that patients with undiagnosed ATTR-CM may have been unintentionally included in these cohorts and equally benefited [18,19]. The recently published observational study by Porcari and colleagues investigated SGLT2i treatment in patients with ATTR-CM, demonstrating a significant association with lower all-cause mortality (HR 0.57, 95% CI 0.37–0.89, *p* = 0.010), lower cardiovascular mortality (HR 0.41, 95% CI 0.24–0.71, *p* < 0.001), and lower rates of hospitalization due to heart failure (HR 0.57, 95% CI 0.36–0.91, *p* = 0.014) in a propensity score-matched cohort [15]. Regarding all-cause mortality, our findings suggest that these associations occur independently of renal function, NT-proBNP levels, systolic function, and concomitant tafamidis therapy. However, accounting for the immortal time bias, any association became neutral in our cohort. This bias has often been neglected in previous observational cohort studies evaluating drug effects—which may have created illusional and overoptimistic treatment effects [20]. In observational studies, treatment may be initiated after baseline examination and during the observational period, indicating a treatment-naïve period of patients within the treatment group. Inherent to the study design, the outcome event cannot occur during this treatment-naïve period so that patients within the treatment group are “immortal” before exposure. Several approaches can be applied to account for this potential immortal time bias [20,21]. An increased awareness of immortal time bias should be applied to all observational cohort studies. Nevertheless, even after accounting for this bias, observational cohorts remain susceptible to other potential biases, especially regarding selection, indication, and confounders. Given the neutrality of our results in context with positive associations observed in larger observational studies, a randomized controlled trial is warranted to better inform about the real effects of SGLT2i in ATTR-CM.

Another possible method to further address the impact of SGLT2i treatment in patients with ATTR-CM might be the usage of large-scale registries of health systems with emulation of randomized controlled trials. In the absence of evidence from classical randomized controlled trials, results from these studies could enhance an understanding of SGLT2i usage in patients with ATTR-CM [22].

An association between SGLT2i treatment and a reduction of atrial fibrillation was described in previous meta-analyses [23]. However, there are conflicting results regarding this association in patients with heart failure, and patients with ATTR-CM were not intentionally represented in these studies.

### 4.1. Strengths and Limitations

A major strength of this study, aside from providing real-world data in a non-prespecified cohort of patients with ATTR-CM, is the clear presentation of the potential immortal time bias. However, there are some limitations to this study. The observational nature of the data, the limited sample size, and the single-centre design may provide effect sizes that are considerably greater than those reported in large-scale trials in heart-failure patients, limiting the generalizability of these results. Further, the lack of racial diversity in our cohort, given the foremost white ancestry in Austria, does not allow for conclusions on patients with African ancestry. This might be of particular interest considering the predominance of hereditary ATTR-CM in these patients, as reported in studies based in the United States, and warrants further investigations [24]. Due to the low number of cardiovascular events, related outcome associations may be subject to a type-2 error. Furthermore, indication bias may have played a role in the introduction of SGLT2i treatment in these patients. Although our findings remained significant after adjusting for confounding variables, we cannot exclude the presence of other confounders not accounted for. 

### 4.2. Conclusions

Patients with heart failure and ATTR-CM represent a high-risk population with continued unmet therapeutic needs. In crude analysis, SGLT2i treatment is associated with better survival in these patients; however, after adjusting for immortal time, this association becomes neutral in our cohort. Hence, to clarify these findings and draw final conclusions on the effectiveness of SGLT2i therapy in patients with ATTR-CM, a randomized controlled trial is warranted.

## Figures and Tables

**Table 1:** Baseline characteristics.

	All	SGLT2i Therapy	SGLT2i-Naïve	*p*-Value
	n = 116	n = 51	n = 65	
Age, years	80 (76–82)	80 (76–82)	80 (77–83)	0.732
Female, n (%)	17 (15)	5 (10)	12 (18)	0.191
Ethnicity Caucasian, n (%)	116 (100)	51 (100)	65 (100)	-
Diabetes mellitus, n (%)	23 (20)	10 (20)	13 (20)	0.958
NYHA-class, n (%)				
I	12 (12)	5 (10)	7 (13)	0.989
II	25 (24)	12 (24)	13 (24)	
II–III	22 (21)	10 (20)	12 (22)	
III	40 (39)	20 (41)	20 (37)	
IV	4 (4)	2 (4)	2 (4)	
Angina pectoris symptoms, n (%)				
Typical	20 (19)	9 (18)	11 (20)	0.708
Atypical	12 (12)	7 (14)	5 (9)	
Tafamidis therapy, n (%)	17 (15)	8 (16)	9 (14)	0.781
Loop diuretic, n (%)	61 (53)	32 (63)	29 (44)	0.327
Thiazide diuretic, n (%)	12 (10)	3 (6)	9 (14)	0.083
Potassium-sparing diuretic, n (%)	41 (35)	25 (49)	16 (25)	0.038
Body mass index, kg/m^2^	24.7 (22.8–26.2)	24.9 (22.6–26.6)	24.4 (22.9–26.2)	0.860
Systolic blood pressure, mmHg	132 (120–147)	129 (118–152)	132 (121–146)	0.902
Diastolic blood pressure, mmHg	78 (73–86)	78 (73–86)	79 (71–85)	0.927
Heart rate, bpm	70 (60–78)	73 (60–81)	70 (61–75)	0.474
LVEF, %	51 (45–57)	49 (43–52)	54 (49–59)	<0.001
NT-proBNP, pg/mL	2845 (1519–5033)	3224 (1949–4738)	2717 (1183–5049)	0.401
hsTrop-T, pg/mL	57 (35–83)	59 (36–87)	56 (31–81)	0.543
Estimated GFR, mL/min/1.73 m^2^	58 (46–69)	58 (39–68)	59 (48–70)	0.478
Creatinine, mg/dL	1.2 (1.0–1.4)	1.2 (1.0–1.6)	1.1 (1.0–1.3)	0.343
CRP, mg/L	2.3 (1.3–4.2)	2.9 (1.3–4.3)	2.0 (1.3–4.2)	0.591

All parameters reported in median (interquartile range) or frequency (percentage). *p*-values derived from Wilcoxon rank–sum tests and Chi-square or Fischer’s Exact tests, as appropriate. Abbreviations: CRP, C-reactive protein; GFR, glomerular filtration rate; hsTrop-T, high-sensitive Troponin T; LVEF, left-ventricular ejection fraction; NT-proBNP, N-terminal pro-brain natriuretic peptide; NYHA, New York Heart Association; SGLT2i, Sodium–glucose co-transporter 2 inhibitors.

**Table 2:** Baseline characteristics at inclusion or treatment initiation.

	All	SGLT2i Therapy	SGLT2i-Naïve	*p*-Value
	n = 116	n = 51	n = 65	
NYHA-class, n (%)				0.258
I	12 (11)	3 (6)	9 (14)	
II	30 (27)	13 (27)	17 (27)	
II–III	18 (16)	5 (10)	13 (20)	
III	50 (44)	27 (55)	23 (36)	
IV	2 (2)	1 (2)	1 (2)	
Tafamidis therapy, n (%)				
At baseline	39 (34)	30 (59)	9 (14)	<0.001
During follow-up	103 (89)	48 (94)	55 (85)	0.107
Loop diuretic, n (%)	71 (69)	42 (82)	29 (56)	0.004
Thiazide diuretic, n (%)	12 (12)	3 (6)	9 (17)	0.071
Potassium-sparing diuretic, n (%)	46 (46)	30 (61)	16 (31)	0.002
Systolic blood pressure, mmHg	133 (120–149)	135 (120–157)	132 (121–146)	0.626
Diastolic blood pressure, mmHg	81 (70–86)	84 (69–90)	79 (71–85)	0.194
Heart rate, bpm	71 (62–78)	73 (64–81)	70 (62–76)	0.168
LVEF, %	51 (44–57)	46 (39–53)	54 (49–59)	0.002
NT-proBNP, pg/mL	3001 (1488–5227)	3384 (1976–6809)	2718 (1183–5050)	0.661
hsTrop-T, pg/mL	57 (35–85)	59 (36–90)	56 (31–81)	0.687
Estimated GFR, mL/min/1.73 m^2^	57 (42–69)	52 (37–64)	59 (48–70)	0.068
Creatinine, mg/dL	1.2 (1.0–1.5)	1.3 (1.0–1.7)	1.1 (1.0–1.3)	0.400

Baseline characteristics at registry inclusion for SGLT2i-naïve patients or at initiation time for patients receiving SGLT2i therapy. All parameters reported in median (interquartile range) or frequency (percentage). *p*-values derived from Wilcoxon rank–sum tests and Chi-square or Fischer’s Exact tests, as appropriate. Abbreviations: GFR, glomerular filtration rate; hsTrop-T, high-sensitive Troponin T; LVEF, left-ventricular ejection fraction; NT-proBNP, N-terminal pro-brain natriuretic peptide; NYHA, New York Heart Association; SGLT2i, Sodium–glucose co-transporter 2 inhibitors.

**Table 3:** Data on clinical outcomes.

	All	SGLT2i Therapy	SGLT2i-Naïve	*p*-Value
	n = 116	n = 51	n = 65	
Outcomes, n (%)				
All-cause mortality	38 (33)	11 (22)	27 (42)	0.023
Cardiovascular death	14 (12)	4 (8)	10 (15)	0.216
WHF hospitalization	32 (28)	18 (35)	14 (22)	0.100
Observation time, years				
All-cause mortality	2.6 (1.7–3.7)	2.7 (2.0–3.7)	2.5 (1.6–3.7)	
Cardiovascular death	3.0 (2.2–4.1)	2.9 (2.0–3.7)	3.0 (2.3–4.2)	
WHF hospitalization	2.0 (1.2–3.2)	1.8 (1.0–3.0)	2.2 (1.4–3.6)	

All parameters reported in median (interquartile range) or frequency (percentage). *p*-values derived from Chi-square or Fischer’s Exact tests, as appropriate. Abbreviations: SGLT2i, Sodium–glucose co-transporter 2 inhibitors; WHF, worsening heart failure.

**Table 4:** Mortality analysis.

	Univariable			Adjusted Model		
	HR	95% CI	*p*-Value	HR	95% CI	*p*-Value
SLGT2i	0.457	0.227–0.922	0.029	0.177	0.062–0.504	0.001
Age				1.023	0.940–1.113	0.598
Sex, male				0.428	0.151–1.217	0.112
eGFR				0.991	0.961–1.022	0.562
NT-proBNP_(log)_				2.164	1.221–3.837	0.008
LVEF				0.991	0.949–1.035	0.689
Tafamidis				1.844	0.794–4.285	0.155

Associations between SGLT2i therapy and all-cause mortality. Multivariable model adjusted for age, sex, and clinically significant confounders. Abbreviations: CI, confidence interval; eGFR, estimated glomerular filtration rate; HR, hazard ratio; LVEF, left-ventricular ejection fraction; NT-proBNP_(log)_, log-transformed N-terminal pro-brain natriuretic peptide; SGLT2i, Sodium–glucose co-transporter 2 inhibitors.

**Table 5:** Cox-regression analysis, including immortal time bias adjustment.

	Univariable			Adjusted Model		
	HR	95% CI	*p*-Value	HR	95% CI	*p*-Value
SLGT2i	1.075	0.524–2.206	0.843	0.839	0.352–1.999	0.692
Age				1.035	0.953–1.123	0.415
Sex, male				0.275	0.101–0.753	0.012
eGFR				1.009	0.980–1.039	0.556
NT-proBNP_(log)_				2.012	1.158–3.495	0.013
LVEF				1.002	0.959–1.048	0.917
Tafamidis				0.979	0.445–2.155	0.959

Associations between SGLT2i therapy and all-cause mortality, including adjustment for immortal time. Multivariable models adjusted for clinically significant confounders. Abbreviations: eGFR, estimated glomerular filtration rate; LVEF, left-ventricular ejection fraction; NT-proBNP_(log)_, log-transformed N-terminal pro-brain natriuretic peptide; SGLT2i, Sodium–glucose co-transporter 2 inhibitors.

## Data Availability

The data underlying this article will be shared on reasonable request to the corresponding author.

## Supplementary Materials

The following supporting information can be downloaded at https://www.mdpi.com/article/10.3390/jcm13195966/s1, Figure S1. Landmark analysis; Table S1. Incidence rates; Table S2. Outcome analysis; Table S3. Outcome analysis with immortal time bias adjustment.
