# Supplementary material for: Impact of SGLT2-Inhibitor Therapy on Survival in Patients with Transthyretin Amyloid Cardiomyopathy: Analysis of a Prospective Registry Study
Source: J Clin Med. 2024 Oct 8;13(19):5966. doi: 10.3390/jcm13195966 (PMC11477643; doi:10.3390/jcm13195966)
Supplement: Supplementary file 1 [file jcm-13-05966-s001.zip › jcm-3167550-supplementary.pdf]

Supplementary Material

Impact of SGLT2-inhibitor therapy on survival in patients with transthyretin amyloid cardiomyopathy: analysis of a prospective registry study

Nora Schwegel [1], Christina Toferer [1], David K. Zach [1], Viktoria Santner [1], Viktoria Höller [1], Jakob Lugitsch [1], Markus Wallner [1], Johannes Gollmer [1], Faisal Aziz [2], Dirk von Lewinski [1], Ewald Kolesnik [1], Klemens Ablasser [1], Andreas Zirlik [1], Harald Sourij [2†] and Nicolas Verheyen [1*†]

1 Division of Cardiology, University Heart Center Graz, Medical University of Graz, Graz, Austria
2 Trials Unit for Interdisciplinary Metabolic Medicine, Division of Endocrinology and Diabetology, Department of Internal Medicine, Medical University of Graz, Graz, Austria
† contributed equally as last authors.
* Correspondence: nicolas.verheyen@medunigraz.at; Tel: +43 (0)316 385 12544; Fax: +43 (0)316 385 13733;

Article - Journal of Clinical Medicine, 2024

Supplementary Table S1. Incidence rates.

	SGLT2i-naïve	SGLT2i therapy
**All-cause mortality**		
Overall incidence rate, *(%)*	16.19 (11.11-23.62)	7.55 (4.18-13.64)
Annual incidence rate, *(%)*		
Year I	13.10 (6.55-26.20)	3.93 (0.98-15.71)
Year II	22.46 (12.44-40.55)	4.52 (1.13-18.07)
Year III	12.41 (4.66-33.07)	17.08 (7.11-41.02)
Year IV	11.23 (2.81-44.91)	0.00 (0.00-0.00)
Year V	30.46 (7.62-121.79)	31.20 (7.80-124.74)
**Cardiovascular death**		
Overall incidence rate, *(%)*	5.00 (2.69-9.29)	2.65 (0.99-7.06)
Annual incidence rate, *(%)*		
Year I	4.70 (1.52-14.56)	1.96 (0.28-13.92)
Year II	5.20 (1.68-16.13)	4.42 (1.11-17.67)
Year III	4.63 (1.16-18.51)	3.13 (0.44-22.21)
Year IV	0.00 (0.00-0.00)	0.00 (0.00-0.00)
Year V	19.48 (4.87-77.89)	0.00 (0.00-0.00)
**Worsening heart failure hospitalization**		
Overall incidence rate, *(%)*	8.99 (5.32-15.18)	16.22 (10.09-26.10)
Annual incidence rate, *(%)*		
Year I	11.76 (5.61-24.67)	15.22 (7.26-31.93)
Year II	8.42 (3.16-22.43)	19.58 (8.79-43.57)
Year III	10.62 (3.43-32.94)	11.82 (2.96-47.27)
Year IV	0.00 (0.00-0.00)	11.39 (1.60-80.85)
Year V	0.00 (0.00-0.00)	40.81 (5.75-89.71)

Overall and annualized incidence rates per outcome. Incidence rates are reported in percentage (95 % confidence interval).

Supplementary Table S2. Outcome analysis.

	univariable			adjusted model		
	**HR**	**95%CI**	**p-value**	**HR**	**95%CI**	**p-value**
**All-cause mortality**						
SLGT2i	0.457	0.227-0.922	0.029	0.177	0.062-0.504	0.001
Age				1.023	0.940-1.113	0.598
Sex, male				0.428	0.151-1.217	0.112
eGFR				0.991	0.961-1.022	0.562
NT-proBNP$_{(log)}$				2.164	1.221-3.837	0.008
LVEF				0.991	0.949-1.035	0.689
Tafamidis				1.844	0.794-4.285	0.155
**Cardiovascular death**						
SLGT2i	1.239	0.654-2.346	0.511	0.213	0.035-1.286	0.092
Age				0.949	0.845-1.066	0.379
Sex, male				0.777	0.149-4.054	0.765
eGFR				0.969	0.920-1.021	0.237
NT-proBNP$_{(log)}$				1.313	0.642-2.686	0.455
LVEF				0.984	0.918-1.054	0.639
Tafamidis				1.707	0.384-7.591	0.482
**Worsening heart failure hospitalization**						
SLGT2i	1.715	0.844-3.484	0.136	0.675	0.235-1.938	0.465
Age				0.973	0.896-1.055	0.504
Sex, male				0.638	0.199-2.044	0.450
eGFR				0.966	0.934-0.998	0.039
NT-proBNP$_{(log)}$				1.353	0.842-2.176	0.212
LVEF				0.984	0.941-1.029	0.479
Tafamidis				1.269	0.501-3.216	0.616

Associations between SGLT2i therapy and clinical outcomes. Multivariable models adjusted for clinically significant confounders. *Abbreviations: eGFR, estimated glomerular filtration rate; LVEF, left ventricular ejection fraction; NT-proBNP(log), log-transformed N-terminal pro-brain natriuretic peptide; SGLT2i, sodium-glucose co-transporter 2 inhibitors.*

Supplementary Table S3. Outcome analysis with immortal time bias adjustment.

	univariable			adjusted model		
	**HR**	**95%CI**	**p-value**	**HR**	**95%CI**	**p-value**
**All-cause mortality**						
SLGT2i	1.075	0.524-2.206	0.843	0.839	0.352-1.999	0.692
Age				1.035	0.953-1.123	0.415
Sex, male				0.275	0.101-0.753	0.012
eGFR				1.009	0.980-1.039	0.556
NT-proBNP$_{(log)}$				2.012	1.158-3.495	0.013
LVEF				1.002	0.959-1.048	0.917
Tafamidis				0.979	0.445-2.155	0.959
**Cardiovascular death**						
SLGT2i	1.796	0.875-3.684	0.110	1.015	0.231-4.453	0.984
Age				0.955	0.851-1.072	0.437
Sex, male				0.530	0.107-2.620	0.436
eGFR				0.985	0.939-1.033	0.541
NT-proBNP$_{(log)}$				1.432	0.693-2.960	0.693
LVEF				1.002	0.935-1.073	0.959
Tafamidis				0.813	0.217-3.040	0.758
**Worsening heart failure hospitalization**						
SLGT2i	2.288	1.066-4.913	0.034	1.745	0.686-4.443	0.243
Age				0.972	0.894-1.057	0.507
Sex, male				0.543	0.174-1.693	0.292
eGFR				0.971	0.941-1.001	0.061
NT-proBNP$_{(log)}$				1.407	0.864-2.292	0.170
LVEF				0.993	0.950-1.038	0.759
Tafamidis				0.960	0.421-2.191	0.923

Associations between SGLT2i therapy and clinical outcomes with consideration of a potential immortal time bias. Multivariable models adjusted for clinically significant confounders. *Abbreviations: eGFR, estimated glomerular filtration rate; LVEF, left ventricular ejection fraction; NT-proBNP(log), log-transformed N-terminal pro-brain natriuretic peptide; SGLT2i, sodium-glucose co-transporter 2 inhibitors.*

Supplementary Figure S1. Landmark analysis.

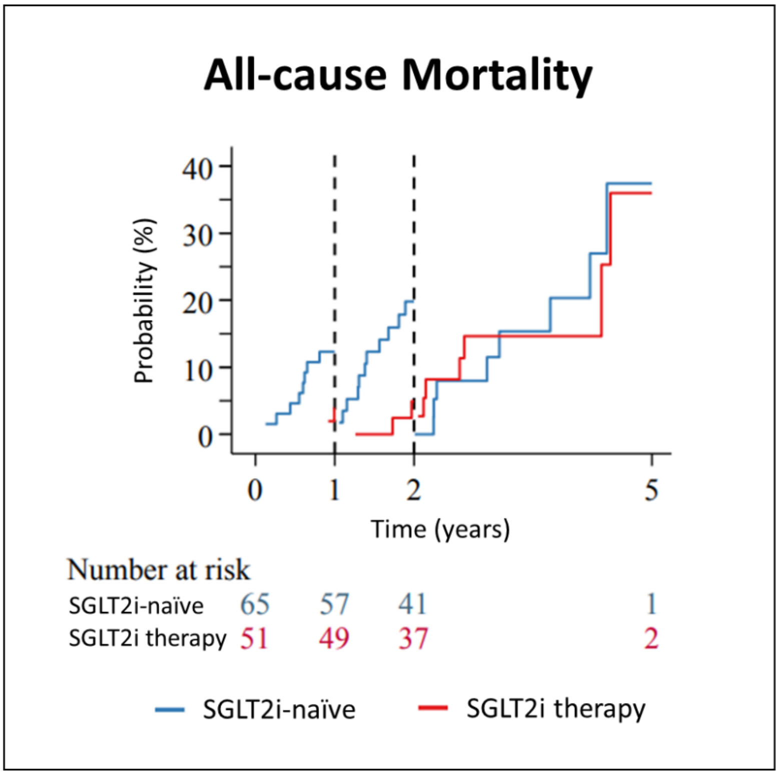

Landmark analysis of survival times for patients receiving SGLT2i-treatment (red) and SGLT2i-naïve patients. The landmark time is set at year one and year two, subjects who survived up to the landmark time are included in the analysis. Survival curves are plotted from the landmark time onwards. *Abbreviations: SGLT2i, sodium-glucose co-transporter 2 inhibitors.*

Stata code sheet.

```
**# Descriptive analysis #1
dtable age i.sex BMI_Baseline BP_sys_Baseline BP_diast_Baseline HR_Baseline /*
*/lvef_baseline Etoeprime_average_Baseline crp_Baseline creatinine_Baseline /*
*/egfr_ckdepi_Baseline tropT_Baseline NTproBNP_Baseline interleukin_6_Baseline/*
*/i.nyha_class_Baseline i.angina_pectoris_Baseline i.hf i.cvdeath /*
*/i.hf_cvdeath i.death,/*
*/by (sglt2i1, tests) sample(, place(seplabels))/*
*/continuous(, statistics(median iqi) test(kwallis))/*
*/define (iqi = q1 q3, delimiter("–")) sformat("[%s]" iqi)/*
*/nformat(%6.2f iqi median) sformat("(%s)" iqi)/*
*/factor(, statistics(fvfrequency fvpercent))/*
*/nformat(%6.2f fvpercent) sformat("(%s)" fvpercent)
dtable i.Tafamidis_yn_atinclusion i.Tafamidis_dailydosis_atinclusion /*
*/i.Tafamidis_yn_atSGLT2i i.Tafamidis_dailydosis_atSGLT2i /*
*/i.NYHA_class_atSGLT2i NTproBNP_atSGLT2i tropT_atSGLT2i /*
*/egfr_ckdepi_atSGLT2i creatinine_atSGLT2i BP_sys_atSGLT2i /*
*/BP_diast_atSGLT2i HR_atSGLT2i EF_echo_atSGLT2i, /*
*/sample(, place(seplabels)) /*
*/continuous(, statistics(median iqi) test(kwallis)) /*
*/define (iqi = q1 q3, delimiter("–")) /*
*/sformat("[%s]" iqi) nformat(%6.2f iqi median) /*
*/sformat("(%s)" iqi) factor(, statistics(fvfrequency fvpercent)) /*
*/nformat(%6.2f fvpercent) sformat("(%s)" fvpercent)
* Describing survival data
tabstat sglt2i_initiat_time hf_time cvdeath_time hf_cvdeath_time death_time, /*
*/s(n mean sd median p25 p75 min max) by(sglt2i1) columns(statistics)
**# Cox regression #2
** All-cause mortality
*Setting-up data
stset death_time1, failure(death==1) id(id)
*Summarizing survival data
strate sglt2i1, per(100)
stsum, by(sglt2i1)
*Log-rank test
sts test sglt2i1
*KM graph
sts graph, by(sglt2i1) failure per(100) risktable /*
*/ytitle("Probability of outcome (%)") xtitle("Time (years)") /*
*/title("A – All-cause mortality") legend(on)
*/title("A – All-cause mortality") legend(on)
*Cox regression
stcox i.sglt2i1
estat phtest
stphtest, detail
stphplot, by(sglt2i1) plot1(msym(oh)) plot2(msym(th))
stcox i.sglt2i1 age i.sex NTproBNP_bl_log egfr_ckdepi_Baseline /*
*/i.Tafamidis_yn_atinclusion lvef_baseline
stphtest, detail
stphplot, by(sglt2i1) plot1(msym(oh)) plot2(msym(th))
stcurve, survival at1(sglt2i1 =0) at2(sglt2i1 =1)
stcurve, hazard at1(sglt2i1 =0) at2(sglt2i1 =1)
stcurve, cumhaz at1(sglt2i1 =0) at2(sglt2i1 =1)
** CV death
*Setting-up data
stset cvdeath_time1, failure(cvdeath==1) id(id)
*Summarizing survival data
strate sglt2i1, per(100)
stsum, by(sglt2i1)
*Log-rank test
sts test sglt2i1
*KM graph
sts graph, by(sglt2i1) failure per(100) risktable /*
*/ytitle("Probability of outcome (%)") xtitle("Time (years)") /*
*/title("C – Composite CV mortality") legend(on)
*Cox regression
stcox i.sglt2i1
estat phtest
stphtest, detail
stphplot, by(sglt2i1) plot1(msym(oh)) plot2(msym(th))
stcox i.sglt2i1 age i.sex NTproBNP_bl_log egfr_ckdepi_Baseline /*
*/i.Tafamidis_yn_atinclusion lvef_baseline
stphtest, detail
stphplot, by(sglt2i1) plot1(msym(oh)) plot2(msym(th))
stcurve, survival at1(sglt2i1 =0) at2(sglt2i1 =1)
stcurve, hazard at1(sglt2i1 =0) at2(sglt2i1 =1)
```

```
stcurve, cumhaz at1(sglt2i1 =0) at2(sglt2i1 =1)
** heart failure hospitalization
*Setting-up data
stset hf_time1, failure(hf==1) id(id)
*Summarizing survival data
strate sglt2i1, per(100)
stsum, by(sglt2i1)
*Log-rank test
sts test sglt2i1
*KM graph
sts graph, by(sglt2i1) failure per(100) risktable /*
*/ytitle("Probability of outcome (%)") xtitle("Time (years)") /*
*/title("D – HF hospitalization") legend(on)
*Cox regression
stcox i.sglt2i1
estat phtest
stphtest, detail
stphplot, by(sglt2i1) plot1(msym(oh)) plot2(msym(th))
stcox i.sglt2i1 age i.sex NTproBNP_bl_log egfr_ckdepi_Baseline /*
*/i.Tafamidis_yn_atinclusion lvef_baseline
stphtest, detail
stphplot, by(sglt2i1) plot1(msym(oh)) plot2(msym(th))
stcurve, hazard at1(sglt2i1 =0) at2(sglt2i1 =1)
stcurve, cumhaz at1(sglt2i1 =0) at2(sglt2i1 =1)
*# Time-depdendent Cox regression #3
** All-cause mortality
*Setting-up data
stset death_date2, failure(death ==1) id (id) scale(365.25) /*
*/origin(registry_date1)
* Replacing sglt2i initiation date with last visit date
replace sglt2i_initiation_date1 = death_date2 + 1 if sglt2i_initiation_date1 ==.
*Splitting survival time
stsplit treatment, at (0) after (sglt2i_initiation_date1)
replace treatment=treatment+1
*Inspecting data
list id _t0 _t _d _st treatment, noobs
*Summarize survival data
strate treatment, per(100)
stsum, by(treatment)
*Log-rank test
sts test treatment
*KM graph
sts graph, by(treatment) per(100) risktable /*
*/ytitle("Survival probability") xtitle("Time (years)")
*Univariate Cox regression
stcox i.treatment
*Cox proportional hazard assumption
stphtest, detail
stphplot, by(treatment) plot1(msym(oh)) plot2(msym(th))
*Multivariable Cox regression
stcox i.treatment age i.sex NTproBNP_bl_log egfr_ckdepi_Baseline /*
*/i.Tafamidis_yn_atinclusion lvef_baseline
*Cox proportional hazard assumption
stphtest, detail
stphplot, by(treatment) plot1(msym(oh)) plot2(msym(th))
*Cumulative hazard curve
stcurve, cumhaz at1(treatment =0) at2(treatment =1)
** CV death
*Setting-up data
stset cvdeath_date2, failure(cvdeath ==1) id (id) /*
*/scale(365.25) origin(registry_date1)
* Replacing sglt2i initiation date with last visit date
replace sglt2i_initiation_date1 = cvdeath_date2 + 1 /*
*/if sglt2i_initiation_date1 == .
*Splitting survival time
stsplit treatment, at (0) after (sglt2i_initiation_date1)
replace treatment=treatment+1
*Listing data
list id _t0 _t _d _st treatment, noobs
*Summarize survival data
strate treatment, per(100)
*Log-rank test
sts test treatment
*KM graph
sts graph, by(treatment) per(100) risktable /*
*/ytitle("Survival probability") xtitle("Time (years)")
*Univariate Cox regression
```

```
stcox i.treatment
*Cox proportional hazard assumption
stphtest, detail
stphplot, by(treatment) plot1(msym(oh)) plot2(msym(th))
*Multivariable Cox regression
stcox i.treatment age i.sex NTproBNP_bl_log egfr_ckdepi_Baseline /*
*/i.Tafamidis_yn_atinclusion lvef_baseline
*Cox proportional hazard assumption
stphtest, detail
stphplot, by(treatment) plot1(msym(oh)) plot2(msym(th))
*Cumulative hazard curve
stcurve, cumhaz at1(treatment =0) at2(treatment =1)
** Heart failure hospitalization
*Setting-up data
stset hf_date2, failure(hf ==1) id (id) scale(365.25) origin(registry_date1)
* Replacing sglt2i initiation date with last visit date
replace sglt2i_initiation_date1 = hf_date2 + 1 if sglt2i_initiation_date1 == .
*Splitting survival time
stsplit treatment, at (0) after (sglt2i_initiation_date1)
replace treatment=treatment+1
*Listing data
list id _t0 _t _d _st treatment, noobs
*Summarize survival data
strate treatment, per(100)
stsum, by(treatment)
*Log-rank test
sts test treatment
*KM graph
sts graph, by(treatment) per(100) risktable /*
*/ytitle("Survival probability") xtitle("Time (years)")
*Univariate Cox regression
stcox i.treatment
*Cox proportional hazard assumption
stphtest, detail
stphplot, by(treatment) plot1(msym(oh)) plot2(msym(th))
*Multivariable Cox regression
stcox i.treatment age i.sex NTproBNP_bl_log egfr_ckdepi_Baseline /*
*/i.Tafamidis_yn_atinclusion lvef_baseline
*Cox proportional hazard assumption
stphtest, detail
stphplot, by(treatment) plot1(msym(oh)) plot2(msym(th))
*Cumulative hazard curve
stcurve, cumhaz at1(treatment =0) at2(treatment =1)
**# Landmark analysis #4
** All-cause mortality
stset death_time, failure(death ==1) id(id)
sts_graph_landmark, at(365 730) by(sglt2i1) risktable end(1825)
** CV death
stset cvdeath_time, failure(cvdeath ==1) id(id)
sts_graph_landmark, at(365 730) by(sglt2i1) risktable end(1825)
** Heart failure hospitalization
stset hf_time, failure(hf==1) id(id)
sts_graph_landmark, at(365 730) by(sglt2i1) risktable end(1825)
*******************************************************************
```
